# Green Breaks: The Restorative Effect of the School Environment’s Green Areas on Children’s Cognitive Performance

**DOI:** 10.3389/fpsyg.2018.01579

**Published:** 2018-10-02

**Authors:** Giulia Amicone, Irene Petruccelli, Stefano De Dominicis, Alessandra Gherardini, Valentina Costantino, Paola Perucchini, Marino Bonaiuto

**Affiliations:** ^1^Department of Psychology of Development and Socialization Processes, Sapienza University of Rome, Rome, Italy; ^2^Department of Human and Social Sciences, Kore University of Enna, Enna, Italy; ^3^Faculty of Economics, Universitas Mercatorum, Rome, Italy; ^4^Department of Nutrition, Exercise and Sports, University of Copenhagen, Copenhagen, Denmark; ^5^CIRPA – Centro Interuniversitario di Ricerca in Psicologia Ambientale, Rome, Italy; ^6^Department of Human Sciences, Society and Health, University of Cassino and Southern Lazio, Cassino, Italy; ^7^Department of Education, Roma Tre University, Rome, Italy

**Keywords:** attention, restoration, nature, school, children, green areas

## Abstract

Restoration involves individuals’ physical, psychological, and social resources, which have diminished over the years in the process of meeting the demands of everyday life. Psychological restoration can be provided by specific environments, in particular by natural environments. Studies report a restorative effect of nature on human beings, specifically in terms of the psychological recovery from attention fatigue and restored mental resources that were previously spent in activities that require attention. Two field studies in two Italian primary schools tested the hypothesized positive effect of recess time spent in a natural (vs. built) environment on pupils’ cognitive performance and their perceived restorativeness, using standardized tests. In Study 1, children’s psychological restoration was assessed by measuring sustained and selective attention, working memory, and impulse control, before and after the morning recess time. Team standardized playtime was conducted in a natural (vs. built) environment, and the perceived restorativeness was measured after each recess time. Results showed a greater increase in sustained and selective attention, concentration, and perceived restorativeness from pretest to posttest after the natural environment condition. In Study 2, the positive effect of free play recess time in a natural (vs. built) environment was assessed during the afternoon school time on sustained and selective attention and perceived restorativeness. Results showed an increase in sustained and selective attention after the natural environment condition (vs. built) and a decrease after the built environment break. Higher scores in perceived restorativeness were registered after the natural (vs. built) environment condition. Team standardized playtime and individual free play recess in a natural environment (vs. built) support pupils’ attention restoration during both morning and afternoon school times, as well as their perceived restorativeness of the recess environment. Theoretical and practical implications are discussed in terms of nature’s role both for the school ground design or redesign and for the organization of the school’s activities.

## Introduction

Restorative environments can be defined as environments that both permit and promote restoration (Hartig, 2004). Restoration refers to the psychological and physiological recovery processes elicited by specific environments and environmental configurations (Joye and Van den Berg, 2011); this recovery process consists of the renewal or recovery of adaptive resources that were depleted in the process of meeting the demands of everyday life (Hartig, 2004). Two main theories—the Attention Restoration Theory (ART) (Kaplan and Kaplan, 1989; Kaplan, 1995) and the Stress Reduction Theory (Ulrich, 1983)—describe the processes underlying the renewal of psychological resources (e.g., the capacity of directing and sustaining attention, inhibiting impulses, and maintaining concentration) through environmental characteristics. Within the scope of the present research, we intend to address psychological restoration elicited by children during break times that are experienced either in a natural or built environment at their school. In particular, we intend to address how children restore their cognitive performance and perceive psychological restoration based on where they spent their recess time—that is, having the break in a natural or a built environment.

This research is grounded in the ART, which is likely to be the most influential theory that investigates the restorative effect of nature on human beings. Specifically, the theory focuses on psychological recovery from attention fatigue and restored mental resources that were previously spent in activities that require directed attention (Kaplan and Talbot, 1983). In fact, directed attention is involved in most daily-life activities; it is voluntary and requires mental effort to ignore distractions and inhibit impulses to maintain focus. Particularly, directed attention is prone to attentional fatigue, which may cause lowered ability to concentrate and solve problems, increased irritability, and increased tendency to make mistakes and incur accidents. Situations that do not require directed attention, allow people to rest the inhibitory mechanism, which eventually leads to attention restoration (Staats, 2012). Within this realm, natural environments help in reducing the constant demands of directed attention; interesting and aesthetically pleasing aspects of natural environments capture attention without an overly high demand for cognitive processing, allowing the mental process known as psychological restoration to occur (Russell, 2012).

In the present research, we aim at two different goals. First, we intend to address children’s cognitive restoration after taking a break (i.e., recess time) in the natural environment or in the built environment of their school. Therefore, we focused on certain attention components subject to depletion during school time. Specifically, we measured working memory, sustained and selective attention, and impulse control, which are the attention components involved in school activities. Second, we intend to address children’s perceived restoration after recess time in the natural or built environment, specifically in terms of the four characteristics that, according to the ART, define a restorative environment (Staats, 2012): people should experience the feeling of *being away*, distant from distractions and demanding stimuli; they should also experience *fascination*, which refers to the person’s effortless attraction for certain environmental elements and engagement in environment-related activities; furthermore, people should sense the environment’s *extent*, which describes its richness and coherence in terms of being perceived as a whole other world; finally, they should feel the *compatibility* between the environment and personal interests, purposes and inclinations, allowing the person to do whatever he/she wants to do. Being away, extent, and compatibility support fascination, which plays a key role in restoration (Kaplan and Kaplan, 1989; Kaplan, 1995). In this study, in addition to the abovementioned three components of attention, we also measured children’s perceived restoration with a self-report scale based on these main characteristics given by the ART. However, in the second experiment, we only measured the most relevant components, *fascination* and *being away* (Gonzalez et al., 2010).

In the literature, several studies have used the ART to understand the restoration processes occurring in everyday life contexts, such as in individuals’ home, school, or workplace. Workplaces, for example, are demanding contexts, and a restorative effect on cognitive capabilities may play an important role on individuals’ well-being and the quality of the work done (Staats, 2012). For example, restorative experiences at the workplace can compensate for job resource demands (Bellini et al., 2015a,b). Some studies, in fact, corroborate the hypothesis of a positive restorative effect of the presence of nature in workplaces: views of nature (compared with other views) reduced stress and increased workers’ job satisfaction (Shin, 2007). Furthermore, workers seem to actively look for contact with nature in their work environment. In offices without windows, people brought indoor plants and pictures of nature (Bringslimark et al., 2011); what followed was an improvement in performance on attention-demanding tasks only from participants in the office with plants (Raanaas et al., 2011) when compared with those in the office without plants. Therefore, for adults, contact with nature seems to be crucial in demanding contexts like workplaces, where they spend most of the day.

Similarly, in everyday life contexts like at home and school, children may experience the same need for restoration provided by nature. Generally speaking, the capacity to direct attention is crucial for children’s everyday activities (Kuo, 2001; Kuo and Sullivan, 2001). Home, for example, is identified as a restorative environment (Hartig, 2012; Wells and Rollings, 2012), and some studies report a positive effect of nature on children’s cognitive functioning (considering children of different ages, up to 18 years). Living in a place with more natural elements can foster children’s improved attentional capacity (Trancik and Evans, 1995; Faber-Taylor and Kuo, 2006), as well as increase their capability to inhibit impulses (Faber-Taylor et al., 2002). Wells (2000) found that, in children between 7–12 years of age, staying at home with an exposure to the natural environment outside is associated with more concentration, attentional capacity, self-discipline, and impulse control. Moreover, specifically for girls the same study reports an association between green view and higher focusing capacity, increased inhibition of impulses, and increased delay of gratification. Flouri et al. (2014) found a connection between near-home nature and less hyperactivity in children, also related to a better emotional resilience and behavioral regulation. In line with this, Wells and Evans (2003) reported an association between the daily at-home contact with nature and stress resilience in children. Moreover, a study conducted amongst children with attention deficit hyperactivity disorder (ADHD) (Faber-Taylor et al., 2001) revealed that exposure to nature through activities carried out in green environments was related to better attentional functioning. In addition, children reported better ratings for activities conducted within natural settings than for activities conducted within built outdoor or indoor settings. In line with these results, self-report measures of parents and caregivers of children suffering from ADHD showed a reduction of symptoms after activities conducted in natural (vs. built) areas (Faber-Taylor et al., 2001; Kuo and Faber-Taylor, 2004; Faber-Taylor and Kuo, 2011). Similarly, a 20 min walk in nature helped ADHD children’s attention capacity (Faber-Taylor and Kuo, 2009), while playing in a natural area helped them to perform better on a concentration task (van den Berg and van den Berg, 2011).

Yet, taking into consideration the environments relevant to children, school is their second main everyday life context. In fact, excluding home, school is where children spend more time than in any other indoor environment (Mendell and Heath, 2005); it certainly is also a cognitively demanding context for them. Therefore, children at school may be in need of restoration and may experience this effect on attention restoration provided by nature. In fact, past research has shown that natural environment in schools helps children to concentrate. Studies have usually compared indoor and outdoor environments (Bagot, 2004; Bagot et al., 2015), focusing either on indoor nature (such as green walls, van den Berg et al., 2016) or on natural views from windows (Liu and Sullivan, 2016). Other studies, on the other hand, have focused on the enhanced working memory and sustained attention of primary school pupils in schools with green and natural surroundings (Dadvand et al., 2015). Moreover, studies on preschoolers have shown that nature may boost children’s concentration (Grahn et al., 1997; Carrus et al., 2015). For example, they are more attentive in areas with trees and shrubbery (Mårtensson et al., 2009); they also express greater attentive abilities and motor coordination in day cares with natural elements (Grahn et al., 1997). Furthermore, natural environments are rated as being more restorative than indoor or built environments when addressing children’s perceived restoration; in particular, comparing more vs. less natural school playgrounds within a given environment was associated to significantly higher perceived restoration (Kelz et al., 2013).

Thus, although research about children’s relationship with nature and with regard to the psychological restoration provided by nature in school environments is indeed increasing (Berto et al., 2015; Dadvand et al., 2015; van den Berg et al., 2016), there is still a research gap regarding some specific aspects. For example, in some instances, a measurement of baseline attention is missing (Berto et al., 2015), or a proper comparison between different outdoor environments belonging to the same school (Bagot, 2004; Bagot et al., 2015; Berto et al., 2015) is yet to be done. However, since school may generate cognitive fatigue, deplete pupils’ resources, and decrease their attention capability (Pellegrini and Davis, 1993), it seems crucial to conduct an assessment of attention restoration on cognitive performance during actual school time. Thus, systematic field studies regarding benefits of nature-at-school on children’s cognitive functioning are still needed. This research aims to fill this gap by addressing these issues with two field experiments conducted within school contexts. Specifically, the present research intends to address whether recess time in a natural (vs. built) environment within the school can provide psychological restoration to a sample of primary school children. Two experimental studies are presented here, which were conducted in the morning and in afternoon school times. An assessment of attention restoration before and after recess in the natural (vs. built) environment and children’s perceived restorativeness of the environments have been provided, and the theoretical and practical implications have been discussed.

### The Research

The current research intends to provide an assessment of children’s psychological restoration after recess time in a natural (vs. built) environment within the school context. In particular, two main general issues are addressed via two field experiments: (a) whether natural (vs. built) environments in schools elicit post break attention restoration in primary scholars and (b) whether pupils perceived the natural environment as more restorative than the built one.

Study 1 investigated whether or not recess time spent in a natural environment at school exerts attention restoration on pupils. The sample was composed of 4th and 5th grade students, in order to compromise between keeping the age as uniform as possible and keeping the procedure manageable with standard tools. Standardized measures of the three attention components, working memory, sustained and selective attention, and impulse control were used. A mixed-model crossover design was used, where the test/retest experiment was conducted in two different outdoor environments of the same school (natural vs. built). By this procedure, it was possible to rule out possible confounds linked to the indoor vs. outdoor distinction. We also used controls for the activity carried out by the pupils during their recess time in order to prevent other confounding effects related to the children’s play; thus, the same team play competitive activity was administered during recess time in both environments. Also, perceived restorativeness was measured with an Italian version of the perceived restorativeness scale (PRS) adapted for children (Hartig et al., 1997; Pasini et al., 2009). This first study focused on the morning school session.

In line with Study 1, Study 2 was developed to address the positive effect of natural (vs. built) environment on attention restoration and perceived restorativeness on primary school children. We made some changes in the procedure and research design in order to address the generalizability of the results produced by Study 1. Specifically, in this between-subjects experiment, we avoided a possible learning effect that could have occurred in the attention scores given that we repeated the same test multiple times. In terms of attention measurement, only one of the three attention tests used in Study 1 was maintained. Children were tested with two measurements of sustained and selective attention (i.e., the main attention dimension involved during school time). Also, we conducted the study during the afternoon-lunch time, assuming that children could be more in need of restoration because they would have accumulated attention fatigue during the morning. Furthermore, during recess time, children were not allowed to engage in any team play activity, but they could play freely in the environment (natural vs. built). The free play was chosen in order to give them the opportunity to truly explore and experience the environment, which, on the contrary, in the previous study was left as the surrounding for a structured team game. Finally, sampled children were from the 5th grade only, since they were easier to manage when compared with the 4th graders, based on the experience from Study 1, within a standard procedure.

## Study 1: Morning Recess Time and Attention Restoration

Study 1 aimed to test the attention restoration provided by a natural environment within the school context when compared with a built one. Thus, a quasi-experimental design assessed the three different attention components involved during school time. Specifically, with a pretest (time 1, T1) and a posttest (time 2, T2) measurement, we tested the positive effect of recess time in natural (vs. built) environment on sustained and selective attention, working memory, and impulse control. Moreover, we addressed children’s perceived restorativeness after recess time in the natural (vs. built) environment, counterbalancing the manipulation order to avoid confounding effects [(a) natural environment condition/built environment condition; (b) built environment condition/natural environment condition]. Then, according to the ART (Kaplan and Kaplan, 1989; Kaplan, 1995), we hypothesized the following.

H1: Children’s sustained and selective attention will be greater in T2 (vs. T1) after recess time in the natural (vs. built) environment.
H2: Children’s working memory will be greater in T2 (vs. T1) after recess time in the natural (vs. built) environment.
H3: Children’s impulse control will be greater in T2 (vs. T1) after recess time in the natural (vs. built) environment.
H4: An interactive effect between condition and manipulation orders on children’s perceived restorativeness can be observed. Specifically, children’s perceived restorativeness will be greater in the natural (vs. built) environment in both the orders’ presentations of manipulation.

### Method

#### Participants and Context

**FIGURE 1:**
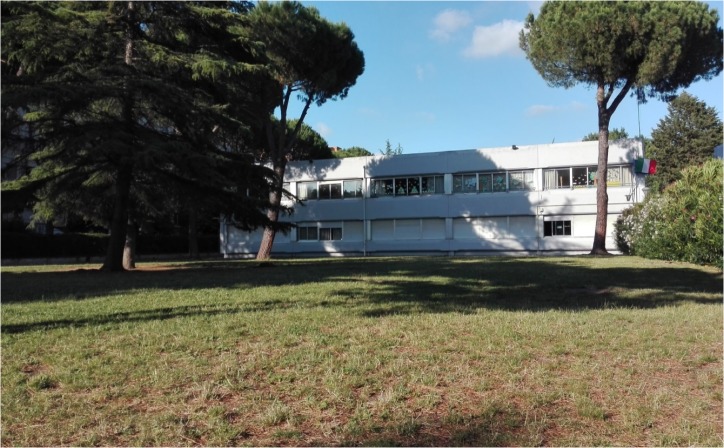
Natural environment of Study 1.

**FIGURE 2:**
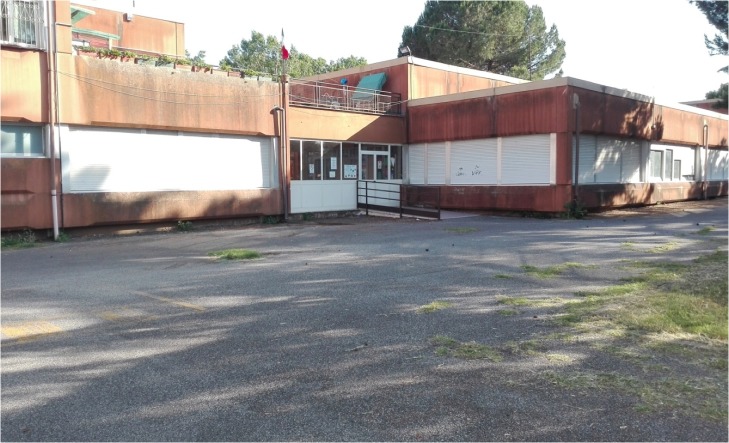
Built environment of Study 1.

The sample was formed by primary school children who attended a public school located in a middle class urban area in Rome, Italy. Eighty-two children (average 10.1 years of age; 39 girls, 43 boys) attending two 4th grade and two 5th grade classes, participated in the study. The school was selected by expert researchers because it offered different outdoor areas, one in a natural environment (**Figure 1**) and one in a built environment (**Figure 2**). The natural area is the school garden (1,303 m^2^), while the built one is the courtyard in front of the school entrance (139 m^2^). We conducted the recess activity either in the whole area of the built environment or in a portion of the school garden resembling the width of the built one, to avoid possible differences that could be derived from playing in a bigger area. Ordinarily, children spend their morning recess time inside their classrooms and the after-lunch break time outdoors (teachers can freely make a decision about bringing them in the natural area or in the built area, which are both known to the children). In this experiment, during the morning recess time, the selected class of children was the only group to play outside during recess time (all other children in the school had the break inside their classrooms as usual).

#### Measures

##### Sustained and selective attention

The Bells test (Biancardi and Stoppa, 1997) is a standardized measure of selective and sustained attention. The test composed of a sheet (21.5 cm × 28 cm) with small black drawings of different symbols (house, tree, bird, bell, etc.). In total, in each sheet there are 35 bells embedded within 280 different distracting stimuli. The attention task involved marking all the bells with a pencil with a time-cap of 120 s. The attention score, ranging from 0 to 35, is calculated based on the total number of bells detected. Wrongly marked symbols are not computed in the final score. The complete test has four different sheets plus a small trial sheet.

##### Working memory

The digit span test (in WISC-IV, Wechsler intelligence scale for children, Fourth edition; Wechsler et al., 2003) is a standardized measure of attention and concentration, which is connected with information maintenance in the working memory. In the original task, administered individually, the person is asked to repeat aloud a progressive series of numbers in the same order as they are given first (digit span forward, DSF) and then in the reverse order (digit span backward, DSB). For the present research, the task was adapted for a collective administration in class; children were asked to listen to the progressive series of numbers and then write down (instead of repeating it aloud) the digit sequence after the “stop” signal displayed by the experimenter. Another experimenter checked if the children did not follow instructions, for example, by writing the sequence before the “stop” signal. Digit span forward is composed of six series of digits (from 2 to 7 digits) and DSB is composed of five series of digits (from 2 to 6 digits). As in the original task, the total score is computed as the sum of the precisely written series (DSF and DSB).

##### Impulse control

The go-no-go test (in BIA, battery for the assessment of children with ADHD, Marzocchi et al., 2010) measures the capacity to inhibit a dominant response. Children receive a marker pen and a sheet (21.5 cm × 28 cm) with 20 items, each item composed a drawing of a “path” made up of 14 squares. The test involved listening and executing the instructions given by a tape. For each item, the tape plays a series of two types of sounds, the “go” tone and the “no-go” tone. The “go” and “no-go” sounds are identical for the first 208 ms, while the no-go tone is marked by a concluding exclamation sound. When the tape begins, children start from the first item (path 1). When they hear the “go” tone, they have to dot the first available square of the path with a marker pen; on the contrary, when they hear the “no-go” tone, they have to inhibit the dominant response of dotting the square and not make the move on the path. The score is calculated based on the number of correct items (paths with the correct number of dotted squares according to the tape) out of 20.

##### Perceived restorativeness

The original self-report scale for measuring perceived restorativeness (PRS) was developed by Hartig et al. (1997). In this study, the Italian short version (Pasini et al., 2009) was used. The scale, comprising of eight items with an 11-point scale (from 0 = “not at all” to 10 = “completely”) was adapted for children by rephrasing few items. Most of the items had verbs in the conditional form, which had been replaced with the indicative form to make them more easily comprehensible. Item number 3 (*“Things and activities that I see there seem to complement in quite a natural way”*) was replaced with item number 23, taken from the Italian complete version of the scale (Pasini et al., 2009) (*“There you can easily see how things are arranged”*). Reliability of the final 8-item scale used in Study 1 was either good or sufficient, especially considering that it is based on a sample administration in primary school children (αNaturalEnvironment = 0.78; αBuiltEnvironment = 0.65).

#### Procedure

A brief description of the study was provided through the informed consent sheet, which was then signed by the school and the parents of each child involved. Three couples of parents did not sign the consent form for their children; thus, these three students participated in the activities of the research but were not included in the sample. Participants with parental consent were excluded if children were absent on the testing day.

Before starting with the experimental procedure, children were enrolled in usual school activities starting at 8:30 a.m. Teachers were told to manage usual class activities as if nothing new would occur. Then, children completed the three paper-and-pencil measures of attention immediately pre (i.e., T1) and post (i.e., T2) their break time, in either one of the two different conditions: play in the natural environment (garden) or in the built (courtyard) environment of their school. Right after the break, they also completed a self-report measure of perceived restorativeness—which referred to the place (natural vs. built area) in which they played during the break time. All tests were collectively administered by giving the relevant instructions and a trial task, to assure a complete understanding from the participants. Children were tested with the same procedure in two different weekdays. For each class, the same weekday was chosen within 1 week for administering the procedure, taking care, as much as possible, of keeping the atmospheric conditions and schooling schedule constant. Data were gathered during the spring in order to have nice weather conditions, in March–April 2014.

**FIGURE 3:**
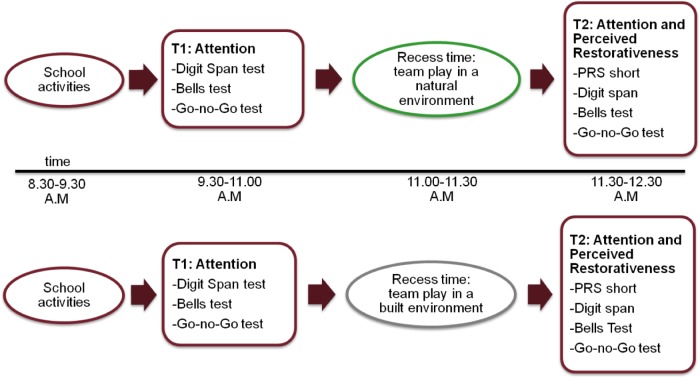
Within-subjects procedure for Study 1.

A within-subjects design was used (**Figure 3**). All children were tested in both the built and natural environment condition during the morning school time. Treatment order was crossed, so that half of the sample (one class from the 4th and one from the 5th grade) was exposed to the natural environment condition first and then to the built environment condition; similarly, the other half of sample was then exposed to experimental conditions in the opposite order.

Children were told to listen carefully to all the instructions and not to cheat. Children’s right to stop the experiment, if they were not comfortable with it, was also clarified. To protect confidentiality, the results of each attention test have been anonymized through a personal identification number assigned to each participant. The procedure followed a given daily timetable and was performed in the morning. Children were asked to play a competitive team-game, after being divided into two teams, with an equal number of girls and boys placed on each team. The activity was a competitive game similar to basketball. Each team consisted of players and a goalkeeper, who held a small wooden stick. The goal of the game was to score points by throwing a rubber ring into the goalkeeper’s stick. Each team had to score on its own goalkeeper and had to prevent the opponent team from scoring as well. The rubber ring could be advanced only with the hands, either by dribbling or by passing to the teammates, moving forward with three or fewer steps. It was forbidden to pass the rubber ring directly to a teammate without throwing it and to take it directly from the opponent’s hands.

In order to test our hypotheses, the presentation order of the two conditions was taken into account as a covariate. In fact, we added the order variable in the procedure to counterbalance the experimental design and to exclude potential confounding effects given by the manipulation of the independent variable. A series of 2 × 2 repeated-measures analyses of covariance (ANCOVAs) were conducted to test the hypothesized significant effect of condition (natural vs. built environment) and time (T1 vs. T2) on sustained and selective attention (H1), working memory (H2), and impulse control (H3), while controlling for the presentation order of conditions. These analyses were followed by a series of protected *t*-tests (Howell, 2012) and by a *z*-test, to allow for the specific hypotheses to be tested. Finally, for PRS, a repeated measures ANCOVA was conducted to test the effect of condition (recess time in the natural vs. built environment) on perceived restorativeness, controlling for the presentation order of conditions (natural-built/built-natural); specifically, we expected a significant main effect of the natural environment condition on perceived restorativeness.

### Results

#### H1: Sustained and Selective Attention

Results (marginal means and standard deviations) for sustained and selective attention are reported in **Table 1**. The repeated-measures ANCOVA showed a significant main effect of condition on the DV controlling for the presentation order, *F*(1,73) = 85.61; *p* < 0.001; $\eta_{p}^{2}$ = 0.54; also, results showed the non-significant effect of time on the dependent variable, *F*(1,73) = 0.13; *p* = 0.72; $\eta_{p}^{2}$ = 0.002. Results showed a non-significant two-way interaction effect of condition and time on sustained and selective attention, *F*(1,73) = 0.18; *p* = 0.67; $\eta_{p}^{2}$ = 0.003. However, we proceeded to the subsequent follow up comparisons through two protected *t*-tests (in line with the recommendations provided by Howell, 2012) in order to test for our specific H1. Results showed that, only in the natural environment, participants significantly restored their sustained and selective attention from T1 to T2. Specifically, when experiencing their recess time in the natural environment, pupils reported a significant improvement in Bells test’s scores from T1 (*M* = 31.85, *SE* = 0.31) to T2 (*M* = 32.61, *SE* = 0.30), *t*(75) = 2.45; *p* = 0.016; *d* = 0.40. Yet, when the recess time occurred in the built environment, participants did not report a significant difference between T1 (*M* = 31.55, *SE* = 0.34) and T2 (*M* = 31.77, *SE* = 0.34) in their sustained and selective attention scores, *t*(75) = 0.73; *p* = 0.47; *d* = 0.12.

**Table 1:** Marginal means, standard deviations and ‘*t*’ of sustained and selective attention, working memory, and impulse control scores in Study 1.

	Sustained and selective attention			Working memory			Impulses’ control		
			
	T1	T2		T1	T2		T1	T2	
Condition	*M* (*SD*; *N*)	*M* (*SD*; *N*)	*t*; sig.	*M* (*SD*; *N*)	*M* (*SD*; *N*)	*t*; sig.	*M* (*SD*; *N*)	*M* (*SD*; *N*)	*t*; sig.
Natural environment	31.85 (2.74; 75)	32.61 (2.70; 75)	*t* = 2.45; *p* = 0.016	15.22 (3.02; 73)	16.38 (3.31; 73)	*t* = 4.12; *p* < ( <0.001	16.85 (3.77; 75)	16.79 (3.66; 75)	*t* = 0.19; *p* = 0.85
Built environment	31.55 (3.59; 75)	31.77 (3.45; 75)	*t* = 0.73; *p* = 0.47	15.42 (3.94; 73)	15.86 (3.27; 73)	*t* = 1.55; *p* = 0.12	16.59 (3.46; 75)	16.97 (2.72; 75)	*t* ( 1.04; p ( =0.30


#### H2: Working Memory

**FIGURE 4:**
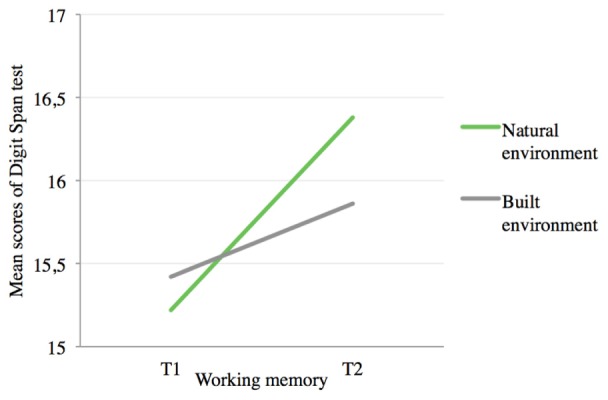
Results of Study 1 for H2. Repeated measures ANCOVA for the three-way interaction effect of condition and time on working memory, controlling for the presentation order [*F*(1,71) = 43.04; *p* < 0.001; $\eta_{p}^{2}$ = 0.38]. Natural environment condition T1 (*M* = 15.22, *SE* = 0.34); natural environment condition T2 (*M* = 16.38, *SE* = 0.38), *t*(73) = 4.12; *p* < 0.001; *d* = 0.68. Built environment condition T1 (*M* = 15.42, *SE* = 0.41); built environment condition T2 (*M* = 15.86, *SE* = 0.38) in their sustained and selective attention scores, *t*(73) = 1.55; *p* = 0.12; *d* = 0.26.

Results (marginal means and standard deviations) for working memory are reported in **Table 1**. The repeated-measures ANCOVA showed a significant main effect of condition on the DV controlling for the presentation order, *F*(1,71) = 21.97; *p* < 0.001; $\eta_{p}^{2}$ = 0.24; also, results showed a non-significant effect of time on the DV, *F*(1,71) = 1.72; *p* = 0.19; $\eta_{p}^{2}$ = 0.02. Importantly, results showed a significant three-way interaction effect of condition and time on working memory, controlling for the presentation order [*F*(1,71) = 43.04; *p* < 0.001; $\eta_{p}^{2}$ = 0.38], which, therefore, was a significant covariate. The subsequent follow up comparisons were conducted through two protected *t*-tests (Howell, 2012), fully confirming H2 (**Figure 4**). In fact, only in the natural environment, participants significantly restored their working memory from T1 to T2. Specifically, when experiencing their recess time in the natural environment, pupils reported a significant improvement in digit span test scores from T1 (*M* = 15.22, *SE* = 0.34) to T2 (*M* = 16.38, *SE* = 0.38), *t*(73) = 4.12; *p* < 0.001; *d* = 0.68. Yet, when the recess time occurred in the built environment, participants did not report a significant difference between T1 (*M* = 15.42, *SE* = 0.41) and T2 (*M* = 15.86, *SE* = 0.38) in their working memory score, *t*(73) = 1.55; *p* = 0.12; *d* = 0.26.

#### H3: Impulse Control

Results (marginal means and standard deviations) for impulse control are reported in **Table 1**. The repeated-measures ANCOVA showed a significant main effect of condition on the DV controlling for the presentation order, *F*(1,73) = 4.33; *p* = 0.04; $\eta_{p}^{2}$ = 0.06; results showed a non-significant effect of time on the DV, *F*(1,73) = 0.60; *p* = 0.44; $\eta_{p}^{2}$ = 0.008. Also, results showed a marginally significant three-way interaction effect of condition and time on impulse control, controlling for the presentation order, *F*(1,73) = 3.73; *p* = 0.06; $\eta_{p}^{2}$ = 0.05. Thus, we proceeded with the subsequent follow up comparisons, conducted through two protected *t*-tests (Howell, 2012), to test H3. Results showed that in the natural environment, participants did not increase their impulse control from T1 (*M* = 16.85, *SE* = 0.43) to T2 (*M* = 16.79, *SE* = 0.42), *t*(75) = 0.19; *p* = 0.85; *d* = 0.03. Neither they did in the built environment, where no difference emerged between T1 (*M* = 16.59, *SE* = 0.40) and T2 (*M* = 16.97, *SE* = 0.31) in their impulse control score, *t*(75) = 1.04; *p* = 0.30; *d* = 0.17.

#### H4: Perceived Restorativeness

**FIGURE 5:**
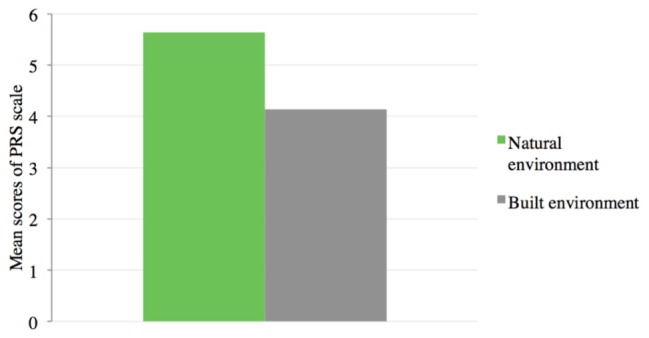
Results of Study 1 for H4. Repeated measures ANCOVA for the effect of condition on perceived restorativeness, controlling for manipulation order, *F*(1,74) = 30.53: *p* = 0.000; $\eta_{p}^{2}$ = 0.292. Natural environment condition (*M* = 5.64, *SD* = 1.59, *N* = 76); built environment condition (*M* = 4.14, *SD* = 2.06, *N* = 76).

Results (marginal means and standard deviations) are reported in **Table 2**. A repeated measures ANCOVA showed a significant main effect of condition on the DV controlling for the presentation order, *F*(1,74) = 30.53: *p* = 0.000; $\eta_{p}^{2}$ = 0.292. Results showed that children rated the natural environment as significantly more restorative than the built one (**Figure 5**).

**Table 2:** Marginal means and standard deviations of perceived restorativeness in Study 1.

Condition	*M* (*SD*; *N*)
Natural environment	5.64 (1.59; 76)
Built environment	4.14 (2.06; 76)


#### Discussion

The results of Study 1 mostly confirmed our hypotheses and provided a series of insights related to the effect of natural environments on the restoration of pupils’ attentive components. After spending their recess time in the natural environment, students performed better both in the sustained and selective attention test and in the working memory test: H1, related to the restorative effect of natural environments on students’ sustained and selective attention, was confirmed at the pairwise comparison level; H2, related to the restorative effect of natural environments on students’ working memory, was fully confirmed; H3, on the other hand, was not confirmed, because no restoration effect was found for impulse control irrespective of the environment in which the students spent their recess time. About this first group of results, it should be noted that our participants performed quite well in all experimental conditions; thus, it is possible that a ceiling effect occurred, buffering the omnibus effect. Accordingly, in Study 2, we slightly modified the test in order to avoid possible learning effects given by the repetition of the exact same test. Finally, H4 was fully confirmed; students reported greater perceived restoration in the natural environment condition (vs. built), controlling for the presentation order. Based on these findings, we proceeded to Study 2, which was conducted following the results and insights that emerged from Study 1. In Study 2, we further explored the effect of the natural environment on students’ attention restoration.

## Study 2: Afternoon Recess Time and Attention Restoration

Study 2 aimed to replicate the significant main effects of Study 1, testing attention restoration provided by a natural (vs. built) environment within the school context while introducing some changes. First, to generalize the attention restoration and perceived restorativeness results with a different procedure, we replaced the crossover design of Study 1 with a between-subjects quasi-experimental design. Furthermore, we conducted Study 2 in the afternoon instead of in the morning because children would have accumulated the full morning load; they would be more tired during the afternoon and perhaps more in need of recovery. Thus, we carried out a pretest (T1) and a posttest (T2) measurement during an afternoon (rather than a morning) school session. Also, during recess time, children were left free to play (contrary to the competitive team play activity rule in Study 1), to test attention restoration effect when children could explore and interact with the environment. These changes were made to allow the broader generalizability of the results. Furthermore, in Study 2, contrary to Study 1, only 5th grade pupils were sampled. Thus, we minimized potential problems related to the management of instructions to be given to two different age-groups of children.

Hypotheses of Study 2 were planned accordingly to the ART (Kaplan and Kaplan, 1989; Kaplan, 1995) and according to the insights provided by Study 1. Here we focused on the measurement of sustained and selective attention only (rather than working memory), as it is more involved in all school activities. Following the results of Study 1 and according to the theoretical basis of the ART, we also hypothesized a higher perceived restorativeness after recess time in the natural environment (vs. built) condition. Therefore, in Study 2 we hypothesized the following.

H5: A significant interaction effect between time (T1/T2) and condition (natural/built) on children’s sustained and selective attention. Specifically, we expect that children’s sustained and selective attention will be higher in the natural (vs. built) environment at T2, whereas no differences are expected at T1 between the natural and built environments.

H6: A greater perceived restorativeness after recess time in children in the natural environment condition (vs. built environment).

### Method

#### Participants and Context

**FIGURE 6:**
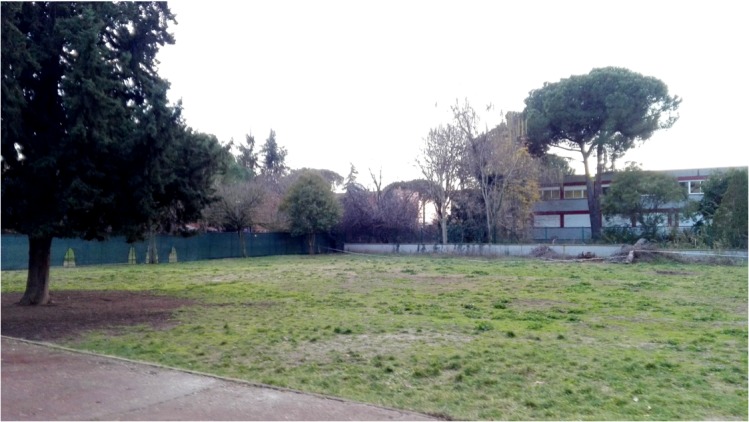
Natural environment for Study 2.

**FIGURE 7:**
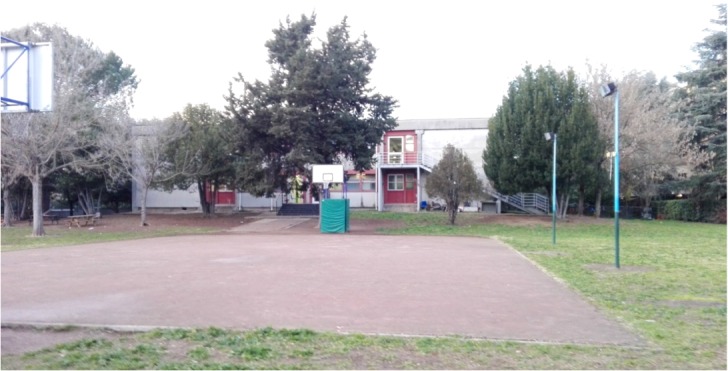
Built environment for Study 2.

The sample was formed by primary school children from a public school located in a middle class urban area in Rome, Italy. Expert researchers selected the school as it offered both a natural area (**Figure 6**) and a built area (**Figure 7**). The two areas used for the experiment have almost equal dimensions (around 460 m^2^) and are close to each other (natural elements are visible from the built area and *vice versa*). Usually, both during ordinary morning and afternoon recess time, children play outdoors, moving freely around both the natural environment and the built environment. Thirty-six children (average 10.8 years of age; 17 girls, 18 boys) participated in the study. Children were recruited from two different 5th grade classes; out of them 18 students, all enrolled in one randomly selected class, were assigned to the natural environment condition; the other 18, enrolled in the other class, were assigned to the built environment condition. During recess time, other children from other classes (not involved in the experiment) were playing outside in the built and natural environments. Similar to Study 1, informed consent was obtained from both the school and the parents; only one student participated in the study activities but was not included in the sample, because her/his parents did not sign the consent form.

#### Measures

##### Sustained and selective attention

As in Study 1, the Bells test (Biancardi and Stoppa, 1997) was used to measure selective and sustained attention. Unlike Study 1, in this study the stimulus to be detected was changed between T1 and T2, in order to diminish any learning effect risk. To keep the tests as comparable as possible to its original version, we selected new target stimuli as they were present in a number similar to the original stimulus; also, the time to detect the new target stimuli was proportioned according to their amount. At T1, the target stimulus was the “bird”: there were 20 birds to be detected in 68 s; alternatively, at T2 the target stimulus was the “house”: there were 21 houses to be detected in 72 s. However, as with the original version of the test, the total number of stimuli in the picture (target stimuli plus distractors) was the same for both the new versions. Wrongly marked symbols were not added to the total score, which ranges from 0 to 20 for T1 and from 0 to 21 for T2. This procedure of using new stimuli in each repetition of the test allowed us to reduce potential confounding effects (e.g., learning effect, boredom, etc.).

##### Perceived restorativeness

The PRS was adapted by the 8-item version used in Study 1 (Pasini et al., 2009). In this study, only the 4 items corresponding to the “fascination” and “being away” constructs were selected, they have been reported as the most relevant ones in terms of perceived restoration (Gonzalez et al., 2010). Reliability of the final 4-item scale was optimal ( =0.80).

#### Procedure

**FIGURE 8:**
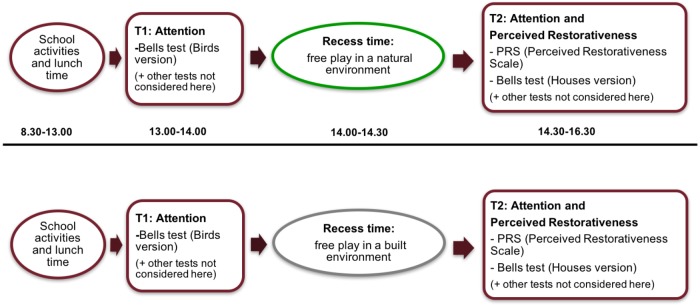
Between-subjects procedure for Study 2.

In Study 2, a between-subject procedure was conducted (**Figure 8**). The sample, which only composed of 5th grade children, was assigned to the two quasi-experimental conditions. The procedure followed a timetable; from 8:30 a.m. to 1:00 p.m., children were enrolled in usual school activities and then had their lunchtime at the school canteen. Students were instructed as described earlier in Study 1; confidentiality and anonymity were assured with the same procedure from Study 1. At T1, from 1:00 p.m. to 2:00 p.m., children performed the attention test (Bells test), other measures have not been considered in this paper. Each one of the tests was administered collectively after a trial administration. After T1, children had their recess time from 2:00 p.m. to 2:30 p.m. (free play in a natural environment vs. built environment); during the 30 min break time, children were told to stay only in the natural (vs. built) environment and that they were free to play whatever they liked. After the break, at T2, from 2:30 p.m. to 4:30 p.m., a drawing task about their break time place was administered (this was not considered in this paper). After the drawing task, the self-report measure of the PRS—which referred to the environment (natural vs. built) they played in—was administered. Then, following the same order administered at T1, the attention test (Bells test) and the other measures were administered. Data were gathered during the end of May 2016; springtime was chosen in order to have a sufficiently milder temperature to comfortably allow outdoor play.

A 2 × 2 mixed model analysis of variance (ANOVA) was conducted to test the interaction effect of condition (natural/built) and time (T1/T2) on selective and sustained attention (H5), measured by the Bells test. Then, a one-way ANOVA was conducted to test the main effect of natural environment (vs. built environment) on perceived restorativeness (H6), measured by the PRS.

### Results

#### H5: Sustained and Selective Attention

**FIGURE 9:**
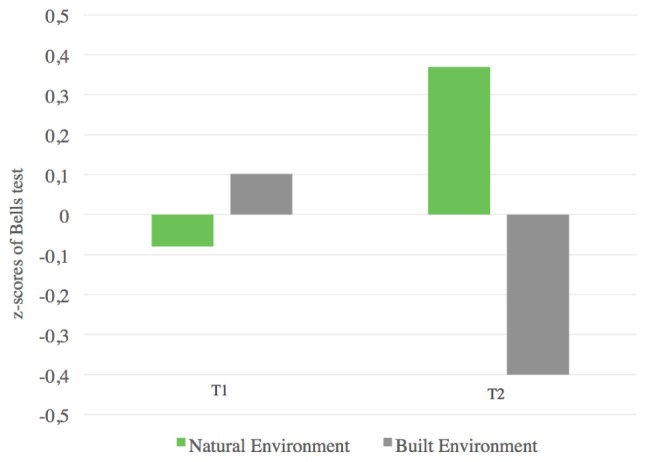
Results of Study 2 for H5. *z*-test for sustained and selective attention scores at T1; natural environment condition (*M* = –0.08; *SD* = 1.21; *N* = 18) and built environment condition (*M* = 0.102; *SD* = 0.78; *N* = 17): *z*(33) = 0.54; *p* = 0.59; *z*-test for sustained and selective attention scores at T2; natural environment condition (*M* = 0.37; *SD* = 1.10; *N* = 18) and built environment condition (*M* = –0.40; *SD* = 0.72; *N* = 17), *z*(33) = 2.47; *p* = 0.007.

Before testing our hypothesis, we standardized the main variables owing to the difference in the two versions of the test used at T1 and T2; by this procedure, the omnibus effect resulting from our main analysis will not be flawed. Results (means and standard deviations) are reported in **Table 3**. A mixed model ANOVA showed non-significant main effects of time^1^ *F*(1,33) = 0.017; *p* = 0.897; $\eta_{p}^{2}$ = 0.001 and condition, *F*(1,33) = 0.983; *p* = 0.329; $\eta_{p}^{2}$ = 0.029. Most importantly, results showed a significant interaction effect of time and condition on the attention score: *F*(1,33) = 10.00; *p* = 0.003; $\eta_{p}^{2}$ = 0.233 (**Figure 9**). Since the main effects were not significant, we proceeded with further analysis given the significant interaction effect and the performed standardization, the specific hypothesized effect was tested with a series of mean difference *z*-tests. Results of the first *z*-test showed, at T1, no significant difference in attention between natural (*M* = -0.08; *SD* = 1.21; *N* = 18) and built environments (*M* = 0.102; *SD* = 0.78; *N* = 17): *z*(33) = 0.54; *p* = 0.59, indicating that pupils sustained and that the selective attention was at the same level before the manipulation occurred. Then, the second *z*-test showed a significant difference at T2; in the natural environment condition, the attention score was significantly higher (*M* = 0.37; *SD* = 1.10; *N* = 18) than in the built environment condition (*M* = -0.40; *SD* = 0.72; *N* = 17), *z*(33) = 2.47; *p* = 0.007, indicating that sustained and selective attention was higher for pupils who spent recess time in the natural environment. On the whole, results confirmed H5 indicating that pupils’ attention was higher after recess time spent in the natural environment than after recess time spent in the built environment. In other words, given the comparable baseline, our results show that sustained and selective attention is better when recess time is spent in the natural environment than when recess time is spent in the built environment.

**Table 3:** Standardized means and standard deviations and *z*-values of sustained and selective attention scores in Study 2.

	Sustained and selective attention		
	
	Natural environment	Built environment	
			
	M (SD; N)	M (SD; N)	z; sig.
T1	-0.08 (1.21; 18)	0.102 (0.78; 17)	*z* = 0.54; *p* = 0.59
T2	0.37 (1.10; 18)	-0.40 (0.72; 17)	*z* = 2.47; *p* = 0.007


#### H6: Perceived Restorativeness

Results (means and standard deviations) are reported in **Table 4**. The one-way ANOVA showed a significant main effect of natural environment (vs. built environment) on perceived restorativeness. Participants reported significantly higher scores in the PRS after recess in the natural area than in the built playground, *F*(1,33) = 10.76; *p* = 0.031, $\eta_{p}^{2}$ = 0.246.

**Table 4:** Means and standard deviations of perceived restorativeness in Study 2.

	Perceived restorativeness
	
	*M* (*SD*; *N*)
Natural environment	5.33 (2.63; 18)
Built environment	2.85 (1.71; 17)


#### Discussion

On the whole, results that emerged from Study 2 further confirmed the attention restoration effect of a natural environment on students’ cognitive performance. The refined measure of sustained and selective attention used here allowed us to overcome the possible limitations that occurred in Study 1; as expected, certain components of attention are better restored if students are allowed to interact with a natural setting than with a built setting. Accordingly, and in line with Study 1, results showed a significant effect of the natural environment (vs. built environment) on perceived restorativeness, confirming that students were reported to feel more restored after spending time in the natural setting of their school.

## General Discussion

The general aim of this research was to investigate the restorative benefits of nature on different cognitive components in children in a school setting, a crucial context of their daily-life. Study 1 produced important new results regarding the attention restoration of different attention components that are typically depleted during school time. In fact, after spending recess time in a natural environment (i.e., at T2), students’ attention scores were significantly higher than the attention scores measured at T1 (before recess time), specifically in terms of sustained and selective attention (H1) and working memory and concentration (H2). Importantly, these effects were not found if students spent their recess time in a built environment. Furthermore, perceived restorativeness (H4) was higher after the natural environment condition (vs. built). Findings from previous studies are in line with our results, both for attention (Wells, 2000; Faber-Taylor and Kuo, 2009, 2011) and perceived restorativeness (Wells, 2000; Bagot, 2004; van den Berg and van den Berg, 2011; Chawla et al., 2014; Bagot et al., 2015; Berto et al., 2015). However, no effects were found on impulse control irrespective of the environmental setting (Schutte et al., 2015), disconfirming H3.

In Study 1, we used a quasi-experimental procedure via a crossover design. This design and the crossed order of the conditions represent controlling factors, which strengthen the interpretation of the results by ruling out some alternative explanation or confounding factors. Other important features of Study 1 consist of the pretest and posttest measurements comparing two school environments that already coexist in a school context; we could keep both the outdoor feature and the activity carried out constant in this study. Therefore, we only manipulated the location of the activity, that is, the crucial environmental feature (natural vs. built) of our study. In this respect, compared to the standard relevant literature, results from our comparisons are noteworthy. Also, it should be noted that these results emerged after the usual study activities conducted in the morning, meaning that our study was realistically embedded in the school routine.

Findings of Study 1 were crucial in designing Study 2 (based on a mixed-model experimental design), in which an interaction effect of time (T1/T2) and condition (natural/built) was hypothesized; specifically, higher attention scores were expected in the natural environment condition at T2. In Study 2, we introduced some changes in the method, in order to rule out potential errors or confounding effects related to the within-subjects procedure used in Study 1: firstly, the experiment was conducted in the afternoon school time, when children may need more restoration; secondly, we used a revised measurement of sustained and selective attention in order to rule out the potential learning effect of repeating the same test more than once (as it was in Study 1); finally, we chose a sample composed of older children (5th grade only) in order to capitalize on their higher ability in instruction comprehension, avoiding other potential confounding factors.

Thus, as expected, Study 2 gave a clearer picture in terms of the anticipated results, by confirming the hypothesized effects. In fact, an interaction effect of condition (natural vs. built) and time (T1 vs. T2) was reported on sustained and selective attention (H5), and a main effect of the natural (vs. built) environment condition was reported on perceived restorativeness (H6); pupils recovered their attention from T1 to T2 after an afternoon break only in the natural setting, and they perceived more restoration after a break in the natural setting (rather than after a break in an equally outdoor but built place). An interesting result was also shown in the built environment condition; in fact, unlike Study 1, attention scores decrease from T1 to T2 in the built environment. As a field study, this result can be better understood if both the characteristics of the environment and the children’s habits during school time are considered. As described earlier, the natural and built environments were close to each other, and during normal recess time children could freely move around both the environments. The instruction to play only in the built environment could have sounded like a limitation for children: they were repeatedly asking the researchers to let them also play in the natural environment. So, playing only in the built environment could have put them in a negative mood, and recess time could not have been restorative for them. This may be identified as a constrained restoration (Hartig et al., 2007), which occurs when the renewal of depleted resource is obstructed by some circumstances (in this case not being allowed to play in the natural environment). One possibility, in further research, should be to investigate the relation between decreased attention in the built environment condition and children’s recess time habits. Moreover, the between-subject design was, in this case, a limitation, providing only one recess time measurement for each condition; in further studies, a crossover design could help to control this effect.

Considering the natural environment condition, results are in line with the findings from Study 1 but also from other studies (Wells, 2000; Faber-Taylor et al., 2001; Bagot, 2004; van den Berg and van den Berg, 2011; Corraliza et al., 2012; Bagot et al., 2015; Berto et al., 2015). Furthermore, the present research showed the positive effect of nature on psychological restoration: (a) in a field study, providing both a crossover and a between-subject design; (b) in a real life situation (our study was embedded in school activities); (c) using preexistent standardized tools for measuring attention involved in school time activities; (d) comparing two different outdoor environments; (e) testing attention restoration in the morning and in the afternoon; (f) assessing attention restoration that occurred after recess time spent in a team play activity and with free play. Specifically, as previous findings have suggested (Bagot et al., 2015), children’s perceived restorativeness experiences during playtime are considered as more important than physical characteristics of the school playgrounds. In literature, the positive effects of nature on attention are reported both when children play in a competitive activity and when they are left free to play (Grahn et al., 1997; Fjørtoft and Sagaie, 2000). In our research, we showed that two equally important activity formats (standardized team play activity in Study 1 and free play in Study 2) are capable of activating the restorative process, provided that a natural (vs. built) outdoor setting is the actual setting for such activities. Moreover, in Study 2 the new 4-item PRS scale (composed of fascination and being away items only) showed optimal reliability (see section “Perceived Restorativeness”). This shorter scale seems more suitable for children, who can be more fatigued by long procedures. Thus, it becomes a useful tool for measuring perceived restorativeness, which is now available for further studies, considering that fascination has been identified as the most important factor in restorative experiences (Staats, 2012).

### Limitations and Future Directions

Although the results that emerged showed a quite clear pattern of effects, some caution should be called in when interpreting such results. First, it should be noted that in Study 1 we might have incurred a ceiling or learning effect due to the repetition of the test materials, which might have buffered the omnibus effect that was found; beyond the significant increase that emerged in the natural environment condition, a slight (yet, non-significant) increase in the attention scores was also registered in the built environment condition. As a *post hoc* speculation, if indeed a ceiling or learning effect occurred in Study 1, this could be related to the version of the go-no-go test. This test, in fact, is normally used to account for attention deficits, and it could not properly detect the small attention fluctuations within the normal population; therefore, it is possible that this specific test, within these conditions and procedure, was simply too easy for the sampled children. Potentially, a between-subjects procedure or a modified test material could solve this issue, as demonstrated in Study 2 where sustained and selective attention was measured by selecting the different sets of target stimuli to be administrated at T1 or T2.

Second, the hypothesized significant increase in impulse control scores (H3) from T1 to T2 in the natural environment condition was unexpectedly unconfirmed (Faber-Taylor et al., 2002; Mårtensson et al., 2009). Although a specific explanation for this unexpected result cannot be drawn from the present research (given the lack of other potential explanation variables), this finding is consistent with recent results showing a similar absence of effect on a similar topic (Schutte et al., 2015). Therefore, further research should deeply focus on the effects of natural environments on impulse control, yet, possibly including mediator or moderator variables, which could eventually explain such specific processes.

Finally, another important point to be discussed is how the children spent their recess time. As highlighted from the present research, nature provides benefits on attention when children are engaged in competitive and fatiguing team play activity (Study 1) and in free play as well (Study 2). In Study 1, children were involved in a team play activity, which were physically and mentally demanding and competitive. On the one hand, by standardizing the break time we controlled for various possible confounding factors, and the activity provided was similar to the usual games children play during recess (e.g., football). On the other hand, though, this team play activity might have reduced the possible restorative effects of nature, because the environment was simply a surrounding background. This arrangement did not match the common relaxing activities usually carried out to elicit attention restoration from natural environments. That is, it did not properly correspond to the prototypical people-environment interaction theorized in the ART for triggering the restorative experience (Hartig et al., 2003). However, results on the perceived restorativeness scores show that children still perceived the differences between the natural and built environments. In fact, even if they did not directly interact with their surroundings with a proper exploration or free play, they rated the experience in the natural surrounding as more restorative than in the built one (keeping constant the time slot, duration, and activity carried out, before and during the break). Potentially, any team activity possessing a proper interaction with the natural environment should, therefore, only increase the restorative effects showed here. Accordingly, in Study 2 recess time was organized to allow children to directly interact with the environment (coherent with the classical restorativeness literature, Hartig et al., 2003). Children were left free to play and explore the environment, engaging in different types of activities and games. This solution overcomes the limits of Study 1 in terms of activity operationalization, thus, avoiding the need to put the environment as just a surrounding background. This, in fact, confirms, and even strengthens, the role of the natural environment in influencing children’s ratings of perceived restorativeness, which were significantly higher after a break in the natural (vs. built) environment condition. However, this can further present a factor that differently impacts the activation of the restorative process; in fact, when children played freely, they could do various activities, and this consequently could elicit multiple variables which are harder to control (e.g., the type of play, the choice of peers to play with, the possibility to have a conflict with a peer, etc.). These variables could have affected the children’s performances measured at T2. Future research could better address these issues, for example by monitoring playtime via videotaping and measuring children’s activity.

On the whole, the decision to assess attention restoration and perceived restorativeness in a quasi-experiment conducted in a field produced both pros and cons. On the one hand, the benefits of nature on children are assessed in their real-life contexts and, so, our results are related to ecologically relevant processes, relations, and activities. On the other hand, various variables are harder to control in a field study than in a laboratory study (e.g., different features of both environments, children’s habits for recess time, normal school activities, etc.). In fact, even if we tried to control for various external factors (e.g., teachers were told to work on the same subject and with a comparable cognitive demand across the various days of administration), certainly there are uncontrollable variables that can interfere with the experiment.

Consequently, taking into account the limitations and findings of the present research program, further studies should assess whether experiences with nature in school settings can affect not only perceived restorativeness and cognitive performance such as attention (as demonstrated in the present study) but also other psychological variables related to children’s experiences at school, such as emotional and affective reactions or social attitudes and behaviors. Furthermore, in the present research (specifically, in Study 1), we ruled out any possible effect related to the order of presentation of our experimental conditions. Yet, we acknowledge that having pupils take their recess time in the built environment first and in the natural environment afterwards (or *vice versa*) could have eventually exerted an effect on their attention restoration and on other related psychological processes; future research should, therefore, investigate such issues. Finally, starting from the proposed experimental procedure, research should develop (quasi-) experiments along new lines, such as integrating psychological measurements with physiological parameters, as some studies already have done (Berto et al., 2015); offering a more precise evaluation of characteristics and dimensions of the tested natural environments (e.g., in terms of presence of natural elements and type of greenery, as described in Bagot et al., 2015); and carefully assessing children’s play and activity features. As Chawla (2015) argued, an optimal option in this field should be the development of an integrated method, using both correlational and experimental designs and ethnographic methods.

## Conclusion

The fundamental importance of providing pupils with school environments that can foster positive learning as well as promoting psychological and physiological well-being is, of course, critical. The ideal school environments seem to be those with an attractive outdoor area, where children can be active both inside and outside of the classroom (Gifford, 2007). Evidence-based design guidelines from research in environmental and architectural psychology should lead to interventions, taking into account children’s needs and contributions in this process (Sanoff and Walden, 2012).

Concrete implications and practical applications should be used for both existing and new school environments, in order to better organize the school’s management and activities by incorporating children’s outdoor natural environments as a crucial feature. In the present manuscript, we provide evidence that natural environments in schools can help students with better recovery of their attention resources, as well as in feeling more restored and less stressed and fatigued. In light of these results, and drawing on the more general literature, we present a set of positive outcomes related to students’ interaction with nature; these outcomes can lead policy makers, schools managers, teachers, and practitioners in general, to promote psychological and physiological well-being of the students in a broader sense.

1. As it has emerged from the present research, after recess time in nature, children in schools show better recovery of their attention abilities and perceive time spent in a natural environment as more restorative than in a built one. This recovery process happens both in the morning and in the afternoon recess time. Literature shows that greener schools help children to concentrate (Bagot, 2004; Bagot et al., 2015; van den Berg et al., 2016) and enhance their attentive abilities (Grahn et al., 1997; Mårtensson et al., 2009).
2. Nature provides benefits for improving attention when children are engaged both in a competitive team play activity and in a free play (Grahn et al., 1997; Fjørtoft and Sagaie, 2000).

Thus, in our view, based on the results that emerged here and in the broader literature on the present topic, green spaces and natural areas should be present in every school; furthermore, they should be used both for leisure and educational activities. Yet, if this is not possible, pupils should have the possibility to get in touch with nature and to engage in activities and learning experiences within natural environments as much as possible, in order to boost their psychological and physiological well-being.

## Ethics Statement

This study was carried out in accordance with the recommendations of the Ethic Committee Guidelines of Sapienza Università di Roma, with written informed consent from all subjects. All participants’ parents gave written informed consent in accordance with the Declaration of Helsinki. The protocol was approved by the Ethic Committee Guidelines of Sapienza Università di Roma.

## Author Contributions

GA, SD, PP, and MB jointly planned the research design and built the procedure. GA, AG, and VC collected data and prepared the data for the analyses. GA and SD carried out data analyses, with the help of feedback from MB. GA drafted the manuscript together with MB, with feedbacks from SD, IP, AG, VC, and PP.

## Conflict of Interest Statement

The authors declare that the research was conducted in the absence of any commercial or financial relationships that could be construed as a potential conflict of interest.

## Footnotes

1 Given that after the standardization the overall mean *z*-score before and after is zero, no main effect of time can be expected.
